# CEBPB, C19MC, and Defective Autophagy Drive Novel Podosomal Belt to Macropinocytosis Transition, Lipid Accumulation, and HBV A-to-I RNA-editing

**DOI:** 10.21203/rs.3.rs-6805130/v1

**Published:** 2025-10-31

**Authors:** Goodwin Jinesh, Nino Mtchedlidze, Varsha Devarapalli, Santanu Adhikary, John Lockhart, Marco Napoli, Isha Isha, Michelle Reiser, Ling Cen, Xiaoxian Liu, Sean Yoder, Tania Mesa, Elsa Flores, Andrew Brohl

**Affiliations:** Moffitt Cancer Center; Moffitt Cancer Center; Moffitt Cancer Center; Moffitt Cancer Center; Moffitt Cancer Center; Moffitt Cancer Center; Saveetha Medical College; Moffitt Cancer Center; Moffitt Cancer Center; Moffitt Cancer Center; H. Lee Moffitt Cancer Center; Moffitt Cancer Center; Moffitt Cancer Center; Moffitt Cancer Center

**Keywords:** Podosomal belt, C19MC, micro RNAs, Hepatitis-B virus (HBV), Chloroquine, Hemin, Mitochondria, Electron Transport Chain, Redox, Macropinocytosis, Vacuole, Lipid droplet, Quinone, CEBPB-LAP, Cirrhosis, obesity, ADAR, RNA-editing, DEPTOR, mTOR, NDUFA3, Lactic acidosis, OXPHOS: oxidative phosphorylation, NICD: Notch1 intracellular domain, NMD: Nonsense -mediated decay

## Abstract

Obesity and neurodegeneration are clinically associated diseases with defective autophagy. However, the genetic, biological, and metabolic underpinnings connecting these diseases are not well-understood. Here we identified a Mitochondria^obesity/neurodegeneration^ (M^on^) gene-signature that is shared between obesity, and neurodegenerative diseases. We demonstrate that, CEBPB elevates M^on^-gene-signature, to form podosomal belts, and enhance ROS production. Inhibiting autophagy collapses podosomal-belts through macropinocytosis to accumulate vacuoles, lipid-droplets, nuclear Notch-1 (nNICD), DEPTOR, and HBV-polymerase mRNAs. Conversely, hemin counteracts these events and suppresses DEPTOR and HBV-polymerase mRNAs by A-to-I-RNA-editing and nonsense-mediated decay. Furthermore, we CRISPR-engineered the antiviral chromosome-19 miRNA cluster (C19MC) to demonstrate that C19MC-miRNAs augment CEBPB, M^on^-gene-signature, ROS, and recapitulate CEBPB-driven phenotypes, in response to autophagy inhibition. Hemin, or a γ-Secretase inhibitor counteract these phenotypes in CRISPR-C19MC-engineered cells. Therefore, a CEBPB and C19MC-driven M^on^-gene-signature regulates the podosomal belt, lipid droplet, HBV, and DEPTOR mRNA dynamics to genetically link obesity, and neurodegeneration at the cellular level.

## Introduction

Obesity, and neurodegenerative disorders such as Alzheimer’s disease (AD), and Parkinson’s disease (PD) are clinically associated metabolic disorders^1–3^. Despite liver metabolism and gut microbiome are implicated in such clinical association, the precise genetic, biological, and metabolic underpinnings connecting these diseases are not well-understood. Obesity, and adipogenesis are regulated at the transcriptional level by CCAAT/enhancer binding protein-β (CEBPB)^4–9^. CEBPB-liver-enriched activator protein (LAP isoform) is the longer isoform of this transcription factor which activates adipogenic obesity transcription whereas the shorter version of CEBPB (liver inhibitory protein: LIP isoform) represses it^9, 10^. Other key cellular regulators of adipogenesis and obesity include the mTOR inhibitor DEPTOR^11^ and increased mitochondrial oxidative phosphorylation (OXPHOS) at liver^12^.

Electron transport-chain system (ETS) is the basic core unit of mitochondria that is organized into complexes I-IV which are located at the matrix side of inner mitochondrial membrane. ETS drives adenosine tri-phosphate (ATP), and reactive oxygen species (ROS) generation through OXPHOS to meet the energy demands of the cell. Despite the genes of ETS components are known for decades, their transcriptional regulation is not yet fully understood. Oxidative damage of mitochondria, its subsequent dysfunction coupled to defective lysosomal clearance or defective mitophagy is a hallmark of both obesity^13^, and neurodegenerative diseases such as Alzheimer’s disease^3^ and Parkinson’s disease^14^.

Mitochondria often get transported to distinct cellular sub-structures for direct delivery of energy. For example, mitochondria localize to neurites/dendrites and non-neuronal filopodia using microtubule-based “+” or “–” end-directed motors. Podosomes are punctate cellular structures that are distinct from filopodia and lamellipodia, that often confused with invadopodia as both these structures are involved in cancer cell invasion and migration^15^. Distinguishing features of podosomes appear when it assembles into a belt surrounding the far outer periphery of the cells but behind lamellipodia, whereas invadopodia usually confined to the plasma membrane location beneath the nuclei^15^. Podosomal belts are capable of forming zipper-like structures to generate multinucleated giant cells^16^ similar to cancer stem cell spheroid formation^17–27^. This spheroid formation is in part due to the podosome’s ability to orchestrate phagocytosis^28^ and endocytosis^29^. Endocytosis and phagocytosis require lipid associated membrane domains, and excessive macropinocytosis^30^ resulting in lipid droplet accumulation^31^ and virus internalization^32^ including Hepatitis-B virus (HBV)^33^. The antiviral chromosome-19 miRNA cluster (C19MC) miRNAs are often coexpressed with CEBPB in cancer^34–37^ characterized against multiple viruses^38^ but not yet examined in the context of HBV. Despite all these factors are related to obesity, metabolism, viral cycle, and neurodegeneration, the precise genetic, metabolic and cell biological underpinnings behind obesity, and neurodegeneration is yet in dark. Therefore, we hypothesized that the obesity driver CEBPB might shed light on the biology of obesity, and neurodegenerative phenotypes at the cellular level and C19MC might have a role in it.

Using human hepatocellular carcinoma (HCC) patient data, and HBV-positive HCC cell line experimentation here we show that, CEBPB-LAP overexpression is tightly linked to Mitochondria^obesity/neurodegeneration^ (M^on^) mRNA expression signature and the core enriched M^on^ genes have CEBPB ChIP-seq binding properties at transcription start sites (TSS). Stable overexpression of *CEBPB-LAP* mimics the CEBPB^High^ tumors by enhancing M^on^ protein NDUFA3 expression, increasing ROS, and forming podosome belts. Inhibiting M^on^ using chloroquine stimulates the accumulation of depolarized mitochondria at podosomal belts, macropinocytosis at podosomal belts to accumulate lipid droplets, increases nNICD, stabilizing DEPTOR and HBV-polymerase RNAs (Podosome-associated phenotypes). Hemin counteracts chloroquine effects and reverses podosome-associated phenotypes. Overexpression of C19MC miRNAs by CRISPR human genetic engineering result in the enhanced M^on^-genesignature along with CEBPB to enhance podosome-associated phenotypes but hemin or γ-secretase inhibitor counteracts podosome-associated phenotypes. Thus, a CEBPB, and C19MC-driven M^on^-gene-signature regulates the HBV and DEPTOR mRNA stability through podosome and autophagy dynamics reflecting HBV viral transcription. Therefore, the CEBPB, and C19MC regulated M^on^-gene-signature genetically links obesity, neurodegeneration, and cancer phenotypes in the context of defective autophagy at the cellular level.

## Results

### CEBPB-LAP promotes the mitochondrial electron transport system to super-charge metabolic phenotypic alterations at the mitochondria, and podosomal belt level

**Figure 1:**
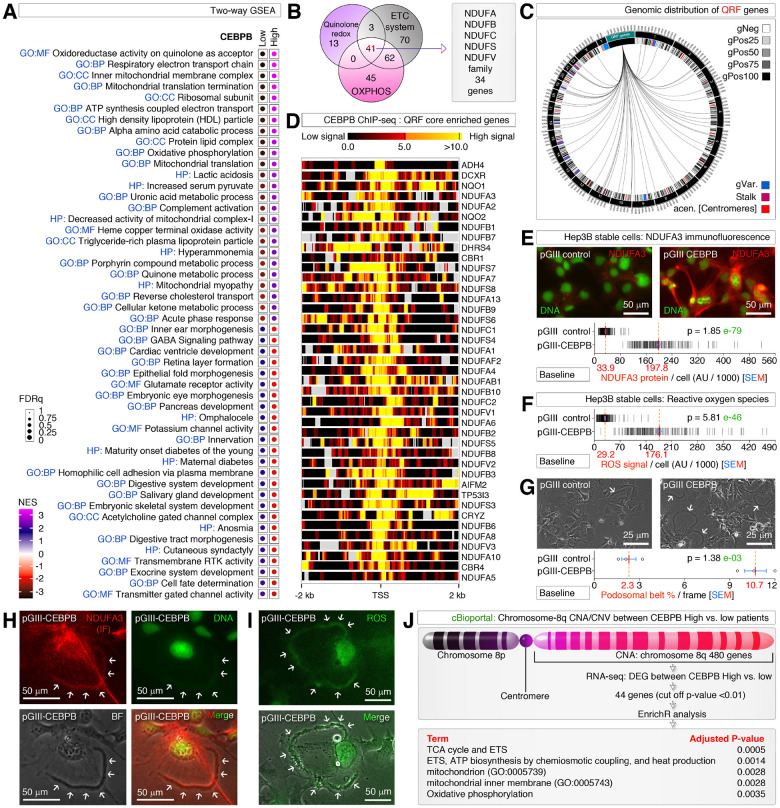
CEBPB-LAP promotes the mitochondrial ETC system to boost the metabolic phenotypic alterations in mitochondria at the podosomal belt level **(A)** Two-way GSEA profiling of CEBPB^High^ versus CEBPB^Low^ hepatocellular carcinomas. **(B)** Multiple metabolic signatures observed in two-way GSEA (Panel-**A**) share a family of NDUF genes. ETC: electron transport chain; OXPHOS: oxidative phosphorylation. **(C)** Genomic distribution of quinolone redox family (QRF) genes showing a genome-wide distribution. gPos or gNeg denotes Giemsa positivity and negativity respectively. **(D)** CEBPB ChIP-seq showing its binding at the TSS of QRF core enriched genes. A 5’−3’ directionality is represented from left to right irrespective of the loci on the positive or negative strand of the genome. **(E)** NDUFA3 immunofluorescence and quantification in CEBPB overexpressed and control cells. pGIII is the lentiviral plasmid construct used for transfections instead of viral infections. AU: arbitrary units. **(F)** Baseline ROS quantification in CEBPB overexpressed and control cells. AU: arbitrary units. **(G)** Baseline podosomal belt formation in CEBPB overexpressed Hep3B and control cells (Arrows). **(H)** CEBPB overexpressed Hep3B cells showing podosomal belt localization of NDUFA3 (Arrows). **(I)** CEBPB overexpressed Hep3B cells showing podosomal belt production of ROS (Arrows). **(J)** Copy number alteration between CEBPB^High^ and CEBPB^Low^ patients showing enrichment of genes from chromosome-8q supporting mitochondrial metabolism.

To understand the gene expression profiles of obesity driver CEBPB^4, 11^ in the liver context, we examined the integrated TCGA-LIHC RNA-seq data{Cancer Genome Atlas Research Network. Electronic address, 2017 #54271} by classifying CEBPB^High^ versus CEBPB^Low^ groups (Figure S1A) and examined the molecular profiles by GSEA. CEBPB^High^ hepatocellular carcinomas (HCCs) exhibit elevated mitochondrial electron transport chain (ETC) system, OXPHOS, mitochondrial complex-I, diabetic, lipid regulatory, and quinolone acceptor redox signatures (Figure 1A). In fact, many of these signatures share a core NADH Dehydrogenase (Ubiquinone) family (NDUF) of genes (Figure 1B) which are well-spread out within the human genome (Figure 1C). Importantly, ChIP-seq data of these genes revealed the presence of strong CEBPB binding sites at the transcription start sites (TSS) and the CEBPB match to the CEBPB-LAP-isoform (Liver-enriched activator protein) (Figure 1D and Figure S1B). We validated this by stably overexpressing CEBPB-LAP in Hep3B cells (Hep3B-pGIII-CEBPB cells) and checked the NDUFA3 protein expression by immunofluorescence to find that a near 6-fold increase in NDUFA3 compared to the control cells (Hep3B-pGIII cells) (Figure 1E). Consistently, we found a ~6-fold increase in ROS production in Hep3B-pGIII-CEBPB cells compared to the Hep3B-pGIII cells (Figure 1F), indicating that not just NDUFA3 but the essential functional ETC system is elevated by CEBPB-LAP. Interestingly, Hep3B-pGIII-CEBPB cells produced ~5 fold more podosomal belts compared to the Hep3B-pGIII cells (Figure 1G). The podosomal belts are positive for NDUFA3 (Figure 1H), and ROS (Figure 1I) indicating the functional mitochondrial localization to podosomal belts. To a lesser extent, sub-chromosomal copy number alteration (CNA) at chromosome-8q also potentially contributes to the increased phenotypic changes in HCC patients (Figure 1J and Figure S1C-D). Taken together, these data demonstrate that a CEBPB-LAP-driven transcriptional change in the elevation of ETC system which super-charges the metabolic phenotypic alterations at the mitochondria and podosomal belt levels.

### Defective autophagy promotes macropinocytosis on podosomal belts to generate large vacuoles which are blocked by hemin

**Figure 2:**
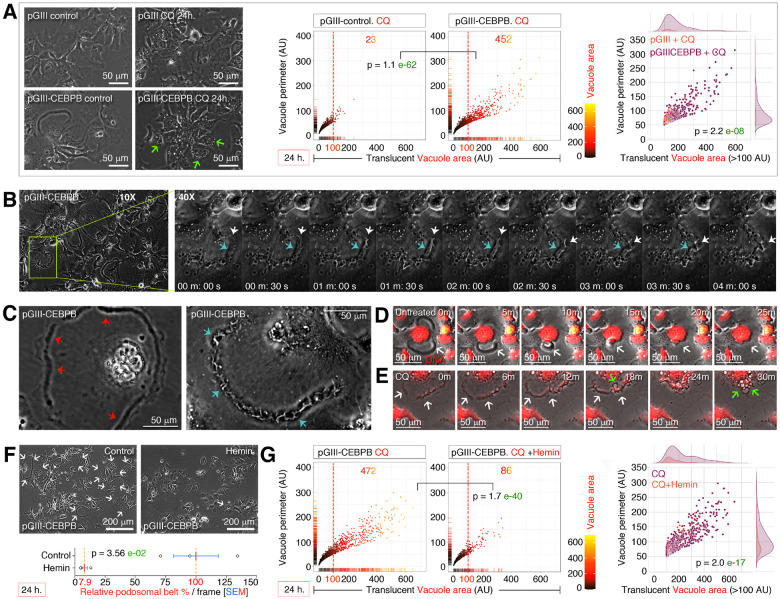
Autophagy inhibition promotes macropinocytosis on podosomal belts to generate large vacuoles, and hemin counteracts it **(A)** Autophagy inhibition with chloroquine (CQ) leads to the loss of podosomal belts and accumulation of translucent vacuoles in CEBPB overexpressed Hep3B cells but not in control cells. AU: arbitrary units. The far-right joint plot uses only the larger translucent vacuoles (>100 AU) from the middle panels. **(B)** Time-lapse microscopy of CEBPB overexpressed Hep3B cell podosome to show the onset of macropinocytic ruffles in the collapse of podosomal belt. Blue arrows show the podosomal belt, and white arrows show the macropinocytic membrane ruffling. **(C)** CEBPB overexpressed Hep3B cells showing stable podosomal belt (Red arrows) and collapsing macropinocytic podosomal belt (Green arrow). **(D)** Time-lapse microscopy of podosomal belt resorption in untreated CEBPB overexpressed Hep3B cells without forming vacuoles (Arrow). Time stamp: in minutes. **(E)** Time-lapse microscopy of podosomal belt (White arrows) resorption in chloroquine (CQ) treated CEBPB overexpressed Hep3B cells forming vacuoles (Green arrows). Time stamp: in minutes. **(F)** Hemin inhibits podosomal belt formation in CEBPB overexpressed Hep3B cells. **(G)** Hemin inhibits CQ-induced podosomal belt collapse and podosomal belt formation in CEBPB overexpressed Hep3B cells to reduce vacuole formation. AU: arbitrary units. The far-right joint plot uses only the larger translucent vacuoles (>100 AU) from the left panels. Note: The smaller <100 AU particles are often artifacts.

Defective autophagy is characteristic feature of obesity^13^, and neurodegenerative diseases^39–41^ and the ETC components are well-known for its ability to act as acceptors for quinolone compounds (Figure 1A) including chloroquine, an autophagy inhibitor^42^. Inhibiting ETC with chloroquine and thus autophagy in Hep3B-pGIII-CEBPB cells resulted in the disappearance of podosomal belts but generated numerous, large (>100 AU), perinuclear translucent vacuoles compared to the Hep3B-pGIII cells (Figure 2A). Using time-lapse microscopy, we discovered that the podosomal belts, in fact, collapse by forming membrane ruffles, and form macropinocytosis vacuoles (Figure 2B). The Non-collapsing versus collapsing podosomal belts can be distinguished by the fleshy podosomal belt morphology versus ruffled podosomal belts, respectively (Figure 2C). The fleshy non-collapsing podosomal belts can also retract itself spontaneously, but it does not generate vacuoles of membrane ruffles in the absence of chloroquine (Figure 2D–E) as evaluated by multiple time-lapse microscopies. While experimenting to understand the role of heme whose oxidase activity was enriched in CEBPB^High^ HCCs (Figure 1A), we discovered that hemin, a chlorinated analogue of heme inhibits podosomal belt formation by >90% (Figure 2F), and thus significantly reduced the number of large translucent vacuole formation in response to chloroquine (Figure 2G). Hemin treated cells also do not display podosomal belts indicating that, the inhibition happens at the level of podosomal belt biogenesis. Taken together these results demonstrate that, defective autophagy promotes macropinocytosis at the podosomal belts to generate large vacuoles which are blocked by hemin through abrogation of podosomal belt biogenesis.

### A mitochondria^obesity/neurodegeneration^ (M^on^)-gene-signature behind podosomal belt reflects macropinocytosis, and lipid droplet accumulation upon autophagy inhibition

**Figure 3:**
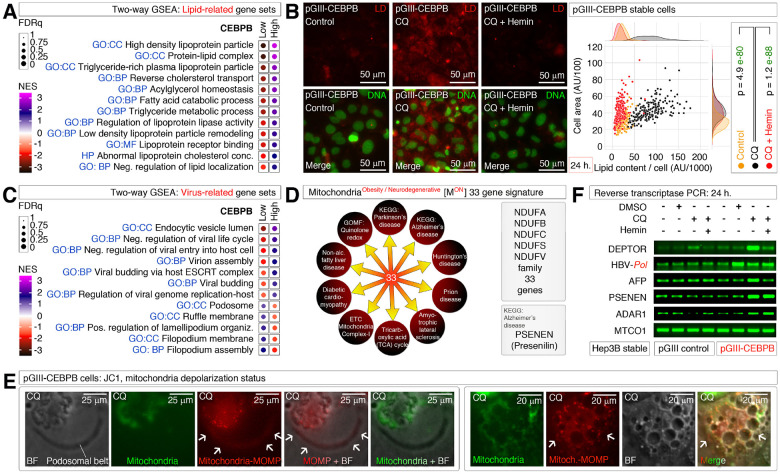
A blockade in mitochondria^obesity/neurodegeneration^ (M^on^)-gene-signature function (autophagy inhibition) drives podosomal belt to lipid droplet accumulation through macropinocytosis **(A)** Two-way GSEA profiling of CEBPB^High^ versus CEBPB^Low^ hepatocellular carcinomas for lipid-related gene sets. **(B)** Lipid droplet staining using Nile Red in CEBPB overexpressed Hep3B cells treated with CQ and or Hemin showing CQ-induced lipid droplet accumulation and is prevented by hemin. The lipid droplet per cell is quantified and plotted on right. AU: arbitrary units. **(C)** Two-way GSEA profiling of CEBPB^High^ versus CEBPB^Low^ hepatocellular carcinomas for virus-related gene sets. **(D)** A 33-gene NDUF family of genes forming a mitochondrial^obesity/neurodegeneration^ (M^on^)-gene-signature. M^on^-gene-signature is shared by multiple neurodegenerative diseases and diabetic and liver pathologies with ETC of obesity. Presenilin gene was from Alzheimer’s disease gene set and not part of the signature (please see downstream text for relevance). **(E)** Accumulation of depolarized (Red: Arrows; MOMP: mitochondrial outer membrane permeabilization) but not polarized (Green) mitochondria at podosomal belts upon chloroquine inhibition (CQ) in CEBPB overexpressed Hep3B cells (Left). Persistence of depolarized mitochondria (Red: Arrows) even after macropinocytosis and vacuole formation in chloroquine treated (CQ) CEBPB overexpressed Hep3B cells (Right). **(F)** RT-PCRs showing selective upregulation of human and HBV-polymerase genes in chloroquine treated (CQ) CEBPB overexpressed Hep3B cells compared to control cells and their suppression by hemin.

Lipid accumulation and adipogenesis are hallmark events of obesity^43^ and lipids are essential for macropinocytosis^32^. In agreement, lipid-related signatures are enriched in CEBPB^High^ HCCs (Figure 3A). Promoting macropinocytosis at podosomal belts of Hep3B-pGIII-CEBPB cells using chloroquine significantly elevated the lipid accumulation, and this phenotype was reversed by hemin by counteracting the chloroquine effects (Figure 3B). Hepatitis B virus (HBV) is known to target lipid transport mechanisms to infect hepatocytes^33^ and most viruses gain entry into the cells through macropinocytosis^32^. Therefore, we examined the virus, and endocytosis-related gene signature enrichments and found that the viral negative regulation signatures, and podosomal signatures were enriched on opposite groups suggesting that, the viral biology might be supported by podosomal activities (Figure 3C).

Importantly, the CEBPB-driven podosomal belt regulatory NDUF gene family (QRF/ETC) shares 33 NDUF-gene members with multiple neurodegenerative diseases such as Parkinson’s disease, Alzheimer’s disease, Amyotrophic lateral sclerosis, prion disease, Huntington’s disease, and non-neuronal diseases like non-alcoholic fatty liver disease, diabetic cardiomyopathy etc. (Figure 3D). We call this CEBPB-driven 33-gene set as mitochondria^obesity/neurodegeneration^ (M^on^)-gene-signature (Figure 3D), which at least in Alzheimer’s disease have a connection to γ-Secretase as inhibitors against this enzyme are aimed for Alzheimer’s disease therapy^44^. We found that the depolarized but not polarized mitochondria localize at podosomal belts and are brought to the core of perinuclear vacuole center upon macropinocytosis (Figure 3E). As the M^on^-gene-signature primarily involves chloroquine sensitive ETC, we continued to experiment its effect on HBV, PSENEN (γ-Secretase gene), DEPTOR (another obesity regulator), ADAR1, an A-to-I RNA-editing enzyme that regulate HBV replication^45^, and DEPTOR mRNA^46^ a γ-Secretase/Notch1/NICD target. We chose HBV-polymerase as a target for evaluation as it controls more HBV-related transcription into an RNA HBV-replication stage despite being a DNA virus. Inhibition of autophagy/M^on^-gene-signature promoted DEPTOR, HBV-pol, AFP (HBV infection: acute phase protein gene), and PSENEN mRNAs in Hep3B-pGIII-CEBPB cells compared to the Hep3B-pGIII cells (Figure 3F). HBV-pol was promoted by DMSO itself (Figure 3F), as it is a known agent promoting HBV replication in hepatocytes^47^. Hemin despite having DMSO as vehicle reduced the HBV-pol mRNA in addition to DEPTOR and AFP mRNAs (Figure 3F). Taken together, these results indicate an ETC/QRF family 33-gene M^on^-gene-signature is behind the podosomal belt to vacuole transition-driven lipid accumulation and its activation upon autophagy inhibition reflects a mechanistic link between obesity and neurodegenerative diseases at the cellular level.

### Podosomal belt dynamics is governed by γ-Secretase, Notch1, mTOR and Src signaling

**Figure 4:**
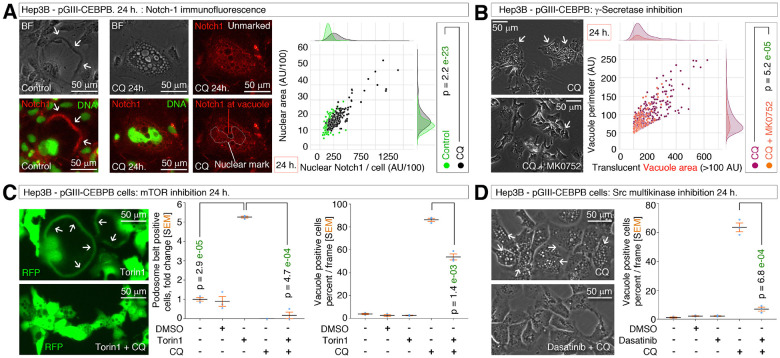
γ-Secretase, Notch1, mTOR and Src signaling regulate podosomal belt dynamics **(A)** Immunofluorescence of Notch-1 showing podosomal belt localization of Notch-1 in untreated control Hep3B CEBPB overexpressed cells (Arrows in left panels) and nuclear localization of Notch-1 NICD. The nuclear boundary is marked by a white line, and an unmarked panel is also included. Quantification of nNICD is represented in the joint plot on the right showing increased nNICD in chloroquine (CQ) treated conditions. AU: arbitrary unit. **(B)** Inhibition of γ-Secretase (using MK0752) blocks podosome to vacuole transition in Hep3B CEBPB overexpressed cells. The translucent vacuole size (>100 AU) and perimeter were quantified and plotted. AU: arbitrary unit. **(C)** Inhibition of mTOR (using Torin1) promotes podosomal belt formation, and chloroquine (CQ) converts those podosomal belts to vacuoles in Hep3B CEBPB overexpressed cells. RFP is pseudo-colored to green. **(D)** SRC inhibition in Hep3B CEBPB overexpressed cells blocks CQ-induced podosomal belt to vacuole transition.

Using the RT-PCR results of chloroquine-treated Hep3B-pGIII-CEBPB cells as clues (Figure 3F), we dissected the podosomal belt dynamics-regulatory events further. DEPTOR and PSENEN (γ-Secretase gene) mRNA expressions are relatable as γ-Secretase activates Notch-1 and activated Notch-1 intracellular domain (NICD) directly known to bind DEPTOR promoter to activate DEPTOR mRNA transcription^48^. Immunofluorescence analysis of Notch1 revealed the localization of Notch1 at podosomal belts in untreated Hep3B-pGIII-CEBPB cells (Figure 4A) and a significant increase in nuclear NICD (nNICD) localization upon chloroquine inhibition of M^on^-gene-signature (Figure 4A). Consistently, γ-Secretase inhibition using MK0752 significantly blocked the vacuole formation from podosomal belts in Hep3B-pGIII-CEBPB cells (Figure 4B) demonstrating the requirement of notch1 activation for macropinocytosis onset at podosomal belts.

DEPTOR transcription downstream to nNICD could have potential effect in macropinocytosis onset at podosomal belts as DEPTOR is an inhibitor of mTOR signaling which regulates amino acid uptake through macropinocytosis. Therefore, DEPTOR protein could have an inhibitory role on macropinocytosis (as in the case of natural retraction of podosomal belts without vacuolization: Figure 2D). We tested this possibility using mTORC inhibitor Torin1. Inhibiting mTORC1/2 complex using Torin1 stabilized podosomal belts without any vacuolization, however the podosomal belts collapsed, underwent macropinocytosis, and vacuolization when Torin1 was cotreated with chloroquine (Figure 4C). Furthermore, inhibiting Src, the upstream regulator of mTOR signaling, using multi-kinase inhibitor dasatinib also blocked the chloroquine-induced podosomal belt to vacuole dynamics (Figure 4D). Therefore, the podosomal dynamics are governed by γ-Secretase, Notch1, mTOR, and Src signaling axis which demonstrates the importance of CEBPB-LAP in promoting DEPTOR mRNA transcription through this axis.

### DEPTOR and HBV-polymerase RNAs are subjected to A-to-I RNA-editing and subjected to nonsense-mediated decay (NMD)

**Figure 5:**
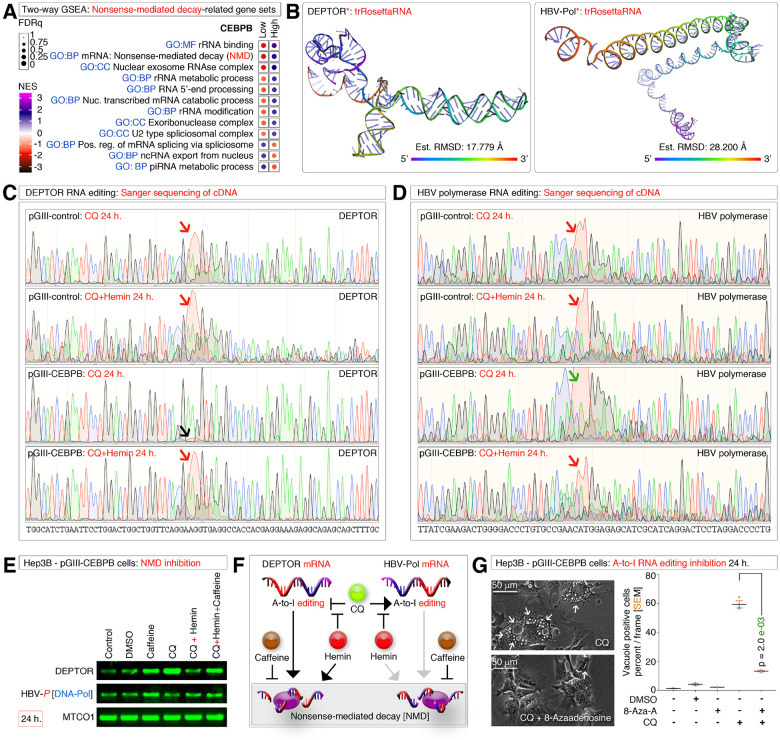
Regulation of DEPTOR and HBV-polymerase RNAs by A-to-I RNA-editing and nonsense-mediated decay (NMD) **(A)** Two-way GSEA profiling of CEBPB^High^ versus CEBPB^Low^ hepatocellular carcinomas for RNA metabolism/nonsense-mediated decay-related gene sets. **(B)** RNA secondary structure prediction of DEPTOR and HBV-pol mRNA regions aimed for amplification from cDNAs. The estimated resolution is indicated. **(C)** Sanger sequencing of cDNAs of DEPTOR showing CEBPB-specific suppression of A-to-I RNA-editing in chloroquine (CQ) treated cells (Black arrow, Red peak) which was restored in CQ + hemin compared to pGIII control cells (Red arrows, Red peaks). Wild-type nucleotide sequence is given below. The window magnifications on both axes were kept constant for comparison purposes. **(D)** Sanger sequencing of cDNAs of HBV-polymerase showing CEBPB-specific promotion of A-to-I RNA-editing in chloroquine (CQ) treated cells (Green arrow, Red peak) which was decreased in CQ + hemin compared to pGIII control cells (Red arrows, Red peaks). Wild-type nucleotide sequence is given below. The window magnifications on both axes were kept constant for comparison purposes. **(E)** Nonsense-mediated decay (NMD) assay of DEPTOR and HBV-polymerase mRNA stability. Note the increase in DEPTOR and HBV-polymerase in caffeine-treated conditions compared to the counterpart controls. **(F)** Schematic showing the effect of NMD on DEPTOR and HBV-polymerase RNAs. Light shaded arrows indicate minor effects than black arrows. **(G)** Evaluation of the effects of A-to-I RNA-editing inhibition using 8-AzaAdenosine (8-Aza-A) on chloroquine (CQ)-induced podosomal belt to translucent vacuole transition in Hep3B CEBPB overexpressed cells.

As DEPTOR and HBV RNAs are known targets for A-to-I RNA-editing by ADAR1^45, 46^ we examined for RNA-regulatory signatures in CEBPB^High^ versus CEBPB^Low^ GSEA results. We found that rRNA (ribosomal RNA) modification and metabolism, RNA-editing, and nonsense-mediated decay signatures are enriched in CEBPB^High^ HCCs (Figure 5A). To examine the RNA-editing we chose novel sites that form RNA secondary structures that aid editing more efficiently in a sequence-based manner (Figure 5B). Sanger sequencing of cDNA amplicons of DEPTOR and HBV-pol mRNAs revealed that both RNAs are differentially edited in Hep3B-pGIII cells versus Hep3B-pGIII-CEBPB cells (Figure 5C–D). For example, under chloroquine-treated conditions, DEPTOR mRNA exhibited RNA-editing in Hep3B-pGIII cells but not in Hep3B-pGIII-CEBPB cells (Figure 5C), explaining why DEPTOR mRNA was found more in Hep3B-pGIII-CEBPB cells compared to Hep3B-pGIII cells treated with chloroquine (Figure 3F). Furthermore, hemin co-treatment with chloroquine could not stabilize DEPTOR mRNA to the level of single agent chloroquine (Figure 3F) because hemin plus chloroquine induced RNA-editing compared to chloroquine alone in Hep3B-pGIII-CEBPB cells (Figure 5C). However, these effects are observed differently for HBV-pol mRNA (Figure 5D), indicating a transcript or site-specific RNA-editing affects the target according to the treatment conditions. Of note, the sites we examined are novel sites of RNA-editing, and there are additional known sites of RNA editing^46^ for these transcripts that when taken into account might determine the overall stability of these RNAs. Therefore, to gain insight into the overall stability of these RNAs, we performed a nonsense-mediated decay (NMD) assay and found that NMD inhibition by itself (using caffeine) stabilized both DEPTOR and HBV-pol RNAs (Figure 5E). However, DEPTOR stabilized better than HBV-pol mRNA under hemin plus chloroquine treated with caffeine when compared to the hemin plus chloroquine treated condition without caffeine (Figure 5E). Therefore, the effect of RNA-editing-mediated NMD effect on HBV-pol RNAs is minimal compared to DEPTOR RNA (Figure 5E–F).

Finally, we examined the effect of A-to-I RNA-editing in the podosomal bet to vacuole dynamics through macropinocytosis by inhibiting RNA-editing using 8-AzaAdenosine (8-AzaA). 8-AzaAdenosine effectively blocked the conversion of podosomal belt to vacuole dynamics (Figure 5G) demonstrating the pivotal role of RNA-editing on DEPTOR, HBV-pol and probable additional targets on podosomal belt dynamics. Therefore, RNA-editing is essential to control podosomal belt dynamics and thus for M^on^-gene-signature effects under defective autophagy conditions.

### CRISPR human genetic engineering to generate a ZNF-331 to C19MC fusion in chromosome-19 to enhance C19MC miRNA expression

**Figure 6:**
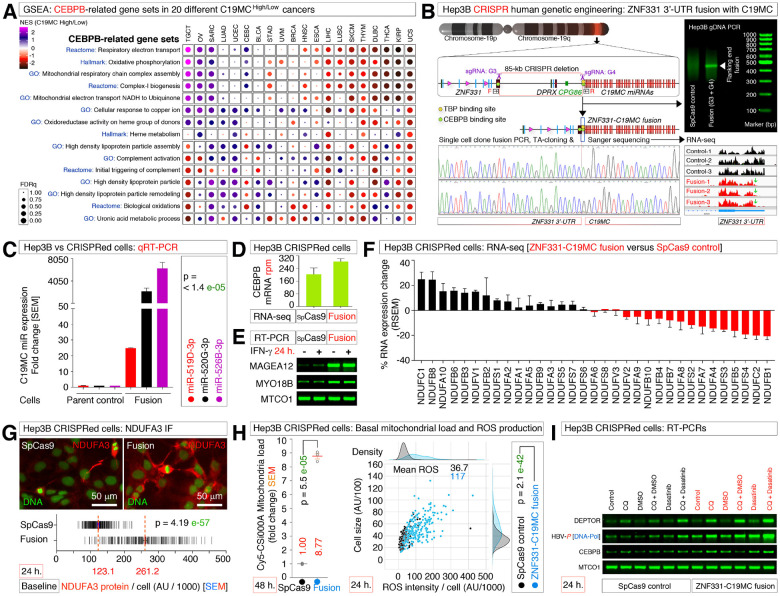
CRISPR-engineered C19MC promotes NDUFA3 protein, ROS, and CEBPB, DEPTOR, and HBV-polymerase RNAs **(A)** GSEA profiling of C19MC^High^ versus C19MC^Low^ 20 different human cancer types showing enrichments of CEBPB-related key gene sets (observed in Figure 1a) in C19MC^High^ cancers. **(B)** CRISPR genetic engineering of human chromosome-19 in Hep3B cells to generate ZNF331-C19MC fusion by deleting 85kb fragment (Red box) using guide-RNAs G3 (cuts at the 3’-UTR end of ZNF331 gene) and G4 (cuts at the start of C19MC) (Purple scissors) and fusing the flanking ends that retain CEBPB binding sites. Flanking end fusion is screened by gDNA PCR (agarose gel inset image) using forward (Fà) and reverse (Rß) primers. Single-cell clones were isolated from SpCas9 control and ZNF331-C19MC fusion Hep3B cells, and Sanger sequenced (chromatogram) to confirm the presence of both fusion and wild-type chromosome-19 in the fusion cells as it exhibits ploidy. The bottom far-right panel shows the RNA-seq reads demonstrating the cut at 3’-UTR of the ZNF331 gene. **(C)** Quantitative real-time PCR showing the ~25-fold upregulation of C19MC miRNAs in Hep3B-ZNF331-C19MC fusion cells. **(D)** RNA-seq data showing the increase in CEBPB mRNA in Hep3B-ZNF331-C19MC fusion cells. **(E)** RT-PCR data showing IFN-γ-independent increase in C19MC target gene mRNAs (MAGEA12 and MYO18B) in Hep3B-ZNF331-C19MC fusion cells. **(F)** RNA-seq data showing a partial increase in M^on^-gene-signature mRNAs in Hep3B-ZNF331-C19MC fusion cells compared to SpCas9 control cells. **(G)** Immunofluorescence showing elevated NDUFA3 protein in Hep3B-ZNF331-C19MC fusion cells. AU: arbitrary units. **(H)** Cy5-CSi000A staining showing elevated mitochondrial load (Left panel) and increased ROS production (Right panel) in Hep3B-ZNF331-C19MC fusion cells. AU: arbitrary units. **(I)** RT-PCR data showing an increase in DEPTOR, HBV-polymerase, and CEBPB mRNAs in Hep3B-ZNF331-C19MC fusion cells in chloroquine (CQ) + Dasatinib combination treated cells.

The involvement of notch-1 activation (nNICD), and its target gene DEPTOR-mRNA enrichment indicated HBV replication, and re-entry cycle though macropinocytosis at podosomal belts, as macropinocytosis is utilized by multiple viruses for entry^32^, and HBV is known to activate notch-1 in liver^49^. This brought our attention to the chromosome-19 miRNA cluster (C19MC), an antiviral miRNA cluster that does not have known effects on HBV yet, but known to co-express with CEBPB in cancer^34^. However, C19MC is known to undergo fusion with other genes in undifferentiated embryonal sarcoma of the liver (UESL) or in pediatric ETMR brain tumors resulting in enhanced C19MC miRNA expression either driven by the fusion partner gene’s transcriptional machinery, or by displacement of the CpG86-island^37, 50–52^. Recently we profiled C19MC associated signatures in 20 different cancer types and used those enrichments to track the CEBPB^High^-associated signatures across 20 different C19MC^High^ cancer types. M^on^-gene-signature-related ETC, and Heme-redox signatures were enriched in a few C19MC^High^ cancer types such as testicular germ cell tumors (TGCT), sarcomas (SARC), and ovarian cancers (OV) but negatively enriched in LIHC without heme redox enrichment (Figure 6A). Therefore, we anticipated a double-edged positive or negative role of C19MC in M^on^-gene-signature function.

To test the role of C19MC in M^on^-gene-signature functionality, we generated Hep3B cells that were CRISPR-engineered to delete an 85-kb segment of chromosome-19 to fuse the 3’-end of 3’-UTR of ZNF331 gene (C19MC’s immediate upstream transcriptionally active gene) with the start site of C19MC while retaining the C19MC proximal CEBPB binding site (Figure 6B). We confirmed the deletion of the 85-kb fragment and fusion of ZNF331-C19MC by three different approaches such as, genomic DNA PCR of flanking ends of 85-kb deleted region, Sanger sequencing of genomic fusion sequence, and RNA-seq (Figure 6B). Of note, Hep3B cells already have basal C19MC expression, and the ZNF331-C19MC fusion resulted in ~25-fold increase in C19MC miRNA expression compared to the parental Hep3B cells (Figure 6C), an increase in CEBPB mRNA expression (Figure 6D) and increased expression of C19MC-regulated target genes MAGEA12 and MYO18B (Figure 6E). Therefore, Hep3B-CRISPR-engineered ZNF331-C19MC fusion is an ideal system to examine the effect of C19MC miRNAs.

### C19MC increases M^on^-NDUFA3 protein, ROS, and CEBPB, DEPTOR, and HBV-polymerase mRNAs.

Analysis of Hep3B-ZNF331-C19MC fusion cell versus Hep3B-spCas9 control cells RNA-seq upregulated gene mRNA data revealed a partial promotion of M^on^-gene-signature at RNA level (Figure 6F). Despite we did not observe RNA level enrichment of complete but partial M^on^-gene-signature, there was a strong and significant accumulation of baseline NDUFA3 at protein level in Hep3B-ZNF331-C19MC fusion cells compared to the control cells (Figure 6G). This result suggests that C19MC miRNAs promote protein translation machinery, as we observed previously^37^. NDUFA3 protein upregulation was accompanied by significantly increased baseline mitochondrial load, and ROS production in Hep3B-ZNF331-C19MC fusion cells compared to the control cells (Figure 6H). These results indicated that the M^on^-gene-signature increase at the protein level is achieved by enhanced expression of C19MC miRNAs, presumably by affecting enhanced translation, or increased protein stability in Hep3B-ZNF331-C19MC fusion cells.

We then tested the effects of chloroquine on M^on^-gene-signature with or without multi-kinase-SRC inhibitor and found that DEPTOR mRNA was promoted by chloroquine to a greater degree in ZNF331-C19MC fusion cells than in SpCas9 control cells (Figure 6I). Likewise, HBV-Pol mRNA was better expressed in ZNF331-C19MC fusion cells than in SpCas9 control cells, in particular at the combination of chloroquine plus dasatinib treated cells (Figure 6I). Importantly, CEBPB was promoted in those same conditions (Figure 6I). Taken together, these data indicate that C19MC increases M^on^-NDUFA3 protein, mitochondrial load, ROS, and CEBPB, DEPTOR, and HBV-polymerase RNAs potentially through the induction of CEBPB.

### C19MC promotes nuclear NICD, lipid accumulation, and RNA editing on Hepatitis B virus (HBV) RNA

**Figure 7:**
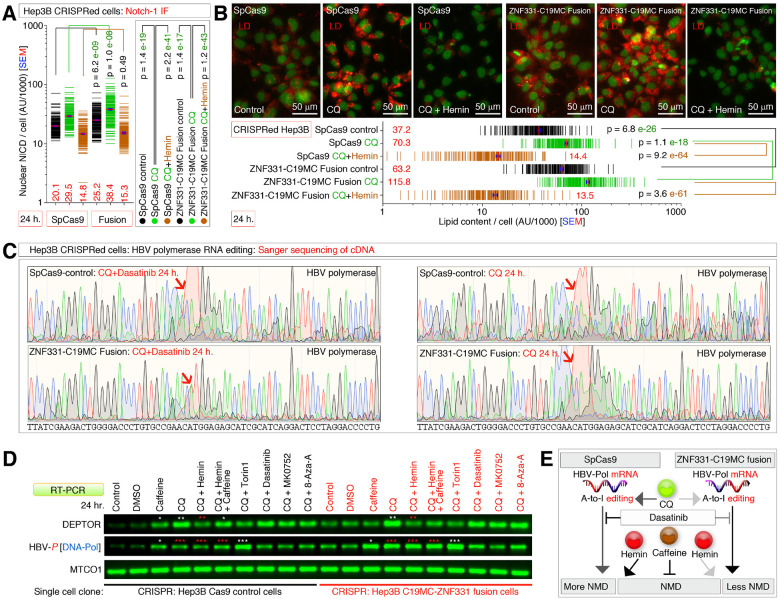
C19MC recapitulates CEBPB effects on nuclear NICD, lipid accumulation, and RNA editing on Hepatitis B virus (HBV) RNA **(A)** Notch-1 immunofluorescence in CRISPR-engineered cells showing increased nuclear NICD (nNICD) in Hep3B-ZNF331-C19MC fusion cells compared to SpCas9 control cells under chloroquine (CQ) treated cells. Note, hemin suppresses chloroquine (CQ)-induced nNICD in both cell types. AU: arbitrary units. **(B)** Nile red lipid droplet quantification in CRISPR-engineered cells showing increased lipid droplets in Hep3B-ZNF331-C19MC fusion cells compared to SpCas9 control cells under chloroquine (CQ) treated cells. Note, hemin suppresses chloroquine (CQ)-induced lipid droplets in both cell types. AU: arbitrary units. **(C)** Sanger sequencing of cDNAs of HBV-polymerase showing ZNF331-C19MC fusion-specific suppression of A-to-I RNA-editing in chloroquine (CQ) + dasatinib treated cells (Red arrows, Red peaks) compared to pGIII control cells. Wild-type nucleotide sequence is given below. The window magnifications on both axes were kept constant for comparison purposes. **(D)** RT-PCRs showing the effect of NMD inhibition (caffeine), mTORC inhibition (Torin1), SRC inhibition (dasatinib), γ-Secretase inhibition (MK0752), and A-to-I RNA-editing (8-AzaAdenosine) in CRISPR-engineered and control Hep3B cells. *, **, ***comparison sets. **(E)** Schematic showing the effect of NMD on HBV-polymerase RNA. Light shaded arrows indicate minor effects than black arrows.

We further examined the key phenotype observed in CEBPB^High^ Hep3B cells that support DEPTOR mRNA expression, such as nNICD translocation. ZNF331-C19MC fusion cells had significantly increased nNICD compared to the SpCas9 control cells at baseline levels, and this difference was further augmented by inhibiting autophagy (chloroquine treated conditions) (Figure 7A). Conversely, hemin suppressed these changes and brought the nNICD levels below the baseline (Figure 7A). Consistent to the nNICD data, the ZNF331-C19MC fusion cells had significantly increased lipid droplet accumulation compared to the SpCas9 control cells at basal levels, and this difference was further augmented by inhibiting autophagy (chloroquine treated conditions) (Figure 7B). Conversely, hemin suppressed these changes and brought the lipid droplet levels below the baseline (Figure 7B). While defective autophagy (Chloroquine treated conditions) and hemin combinations in CRISPR-C19MC-engineered cells behaved similar to CEBPB^High^ versus CEBPB^Low^ cells, the combination of dasatinib plus chloroquine in promoting HBV-pol mRNA in ZNF331-C19MC fusion (Figure 6I) was different. Therefore, we examined the RNA-editing of HBV-pol mRNA and found that, the HBV-pol mRNA was subjected to lesser RNA-editing upon chloroquine plus dasatinib treatment in ZNF331-C19MC fusion cells compared to single agent chloroquine treated ZNF331-C19MC fusion cells or its SpCas9 control counterpart (Figure 7C). We further examined the relative mRNA levels of DEPTOR and HBV-pol and found that, a DEPTOR versus HBV-pol transcript-specific changes in NMD was observed between ZNF331-C19MC fusion cells and SpCas9 control cells in single agent caffeine treated cells (HBV-pol was rescued more than DEPTOR mRNA in fusion cells) (Figure 7D). Furthermore, under hemin, or torin1-treated conditions HBV-pol mRNA expression was more in ZNF331-C19MC fusion cells than in SpCas9 cells compared to the DEPTOR mRNA (Figure 7D–E). Finally, caffeine rescued more HBV-pol mRNA in SpCas9 cells treated with chloroquine plus hemin than in ZNF331-C19MC fusion cells to indicate that HBV-pol RNA undergoes lesser NMD in fusion cells (Figure 7D–E).

Taken together, these data shows that C19MC promotes nNICD, lipid accumulation, and transcript-specific RNA-editing on DEPTOR and Hepatitis B virus (HBV) mRNAs.

## Discussion

Obesity, and neurodegeneration are clinically associated diseases where obesity sets in very early in life compared to the neurodegenerative diseases. However, the central mechanism or biology that connects these diseases at cellular level is elusive to date despite defective autophagy has been identified at hepatic, and neuronal sites^13, 40^. Here we used the obesity driver CEBPB-LAP isoform as the starting point of our investigation in HCC context as this mRNA is most enriched and active in liver. Importantly, we identified that the CEBPB-LAP places itself upstream of Notch1 and DEPTOR signaling nodes which are additionally implicated in obesity and adipogenesis^48^.

**Figure 8:**
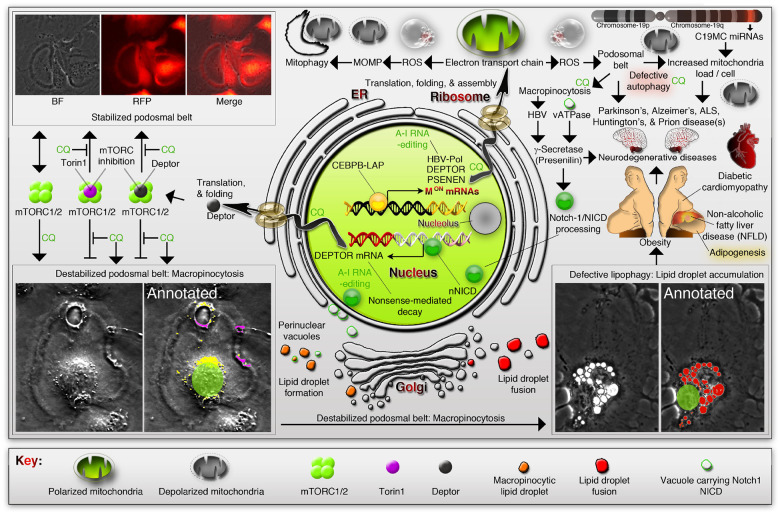
CEBPB and C19MC link obesity and neurodegenerative diseases at the cellular level by regulating M^on^-gene-signature, podosomal belt dynamics through macropinocytosis, lipid accumulation, and RNA-editing upon defective autophagy Schematic showing the DNA binding and transcriptional activation by CEBPB-LAP enhancing M^on^-gene-signature genes which upon translation and folding form electron transport chain within mitochondria resulting in enhanced ROS production and mitochondrial dysfunction such as MOMP. Defective autophagy (Chloroquine: CQ) at this setting results in the accumulation of such mitochondria, that localize to podosomal belts and promote macropinocytosis at podosomal belts to resorb podosomal belts (Bottom left photomicrograph/Destabilized podosomal belt: annotated right panel shows green: nucleus; yellow dots: mitochondria; purple shade: macropinocytic membrane ruffles. These can be compared in an unannotated panel on left). Macropinocytosis serves to internalize lipid droplets (Orange vesicles), which fuse at the trans-Golgi network (TGN) area to form lipid droplets (Red vesicles) (Bottom right photomicrograph/Defective lipophagy: annotated right panel shows, red: lipid droplets at TGN which appear as translucent vacuoles in the unannotated left panel). Lipid droplet accumulation forms the basis for obesity, adipogenesis, and non-alcoholic fatty liver disease. Defective autophagy in turn promotes DEPTOR and HBV-polymerase transcription. HBV and vATPase are capable of triggering γ-Secretase to activate Notch-1 NICD cleavage. Nuclear NICD (nNICD) then promotes DEPTOR mRNA transcription to modulate mTORC1/2 signaling to further regulate podosomal belt dynamics. On the other hand, C19MC promotes mitochondrial load/cell and upon defective autophagy (CQ), promotes lipid accumulation and nNICD accumulation to promote DEPTOR and HBV-polymerase transcription. A-to-I RNA-editing acts at the level of DEPTOR, and HBV-polymerase RNA stability level through nonsense-mediated decay. This schematic links obesity to multiple neurodegenerative diseases at the cellular level through the M^on^-gene-signature, which is the core of all observed phenotypes.

Several evidences from our study demonstrated how the obesity driver CEBPB-LAP is promoting the mitochondrial M^on^-gene-signature and how it is related to multiple neurodegenerative diseases and their biology at the cellular level. First, we identified the mitochondrial electron transport chain (ETC) components as M^on^-gene-signature which is a 33-NDUF-gene mRNA expression signature that is shared by obesity, non-alcoholic fatty liver disease, diabetic cardiomyopathy, and multiple neurodegenerative diseases including Parkinson’s disease, Alzheimer’s disease, Huntington’s disease, and amyopathic lateral sclerosis (ALS) (Figs. 3D and 8). Second, we demonstrated that CEBPB-LAP drives podosomal belt biogenesis and that the NDUFA3 is indeed localized to it with ROS production at podosomal belts (Fig. 1H–I). Elevated ROS confirms that not just NDUFA3 but the essential ETC signature is up and functional in response to CEBPB-LAP, or C19MC (Figs. 1F and 6H). Third, we demonstrated that the quinolone (chloroquine) acceptor usage mimics the defective autophagy (and hence mitophagy and lipophagy) to result in the accumulations of depolarized mitochondria, and lipid droplets, to form a mechanistic link between obesity and neurodegenerative diseases at the cellular level. Notably, these diseases exhibit defective autophagy^13, 40, 41^ (Fig. 8). Fourth, CEBPB-LAP promotes robust lipid accumulation which supports the context of obesity, adipogenesis and endocytosis/macropinocytosis. Fifth, identification of macropinocytosis at the podosomal belt is a novel striking finding that links lipid intake and its accumulation at vacuoles (Fig. 8). Macropinocytosis is the major entry point of numerous human pathogenic viruses^32^. Our study shows that CEBPB promotes RNA stage of HBV replication, and promotion of macropinocytosis upon autophagy inhibition suggests a viable cycle of HBV production and re-entry. More support for this notion comes from the γ-Secretase inhibitor-mediated abrogation of macropinocytosis-to-vacuole transition (Fig. 8) because HBV is known to activate notch1 signaling through γ-secretase activity^49^. Alternatively, vacuolar vATPase could drive notch1 activation^53^ and we also observed vacuolar localization of notch1 (Fig. 4A and Fig. 8). Similar lipid droplet vacuole formation is known in the context of macrophage foam cell formation through macropinocytosis^54^ and we tested THP1-derived macrophages for lipid accumulation in response to chloroquine treatment and confirmed similar lipid droplet accumulation as to CEBPB^High^ or ZNF331-C19MC fusion cells (Data not shown). Finally, using torin1 and chloroquine we demonstrated how mTOR signaling and DEPTOR transcription play a crucial role in podosomal belt to macropinocytosis dynamics in the context of cellular lipid droplet accumulation and defective autophagy (Fig. 8).

Our experiments on C19MC miRNA promotion by CRISPR genetic engineering recapitulates the major phenotypes driven by CEBPB-LAP, promotes HBV transcripts in response to clinically used drugs like chloroquine, dasatinib, and increases the mitochondrial load in cells to reflect defective mitophagy (Fig. 8).

Finally, our study sheds more light on the modulation of A-to-I RNA-editing in a transcript specific manner. We identified novel sites of A-to-I RNA-editing in DEPTOR, and HBV-pol mRNAs and show that, RNA-editing may act in a transcript specific manner in CEBPB-LAP versus C19MC expression context. Congruent to this notion, C19MC^High^ signatures displayed similar as well as contrasting enrichments related to CEBPB-related signatures, particularly the ETC signature which includes M^on^-gene-signature, and the heme-related signature (Fig. 6A).

Taken all the data together, Our study identified an important master switch, the CEBPB-LAP isoform, which drives M^on^-gene-signature, that connects the cellular phenotypes (lipid accumulation, and mitochondria accumulation by defective autophagy) of obesity to neurodegenerative diseases, non-alcoholic fatty liver disease, and diabetic cardiomyopathy.

## Materials and Methods

### Lead contact

Further information and requests for details on materials should be directed to and will be fulfilled by the lead contact, Dr. Goodwin G. Jinesh (goodwinjinesh@gmail.com).

### Materials availability

Further information and requests for materials should be directed to and will be fulfilled by the corresponding author contact, Dr. Andrew S. Brohl (Andrew.Brohl@moffitt.org).

### Data and code availability

This paper does not report original codes but uses modified codes, which are given under the appropriate methods sections below. This paper uses public TCGA data sets, and details are given below under the appropriate methods sections.

### Human subject statement

This study does not deal with human subjects or PHI other than using deidentified TCGA transcriptomics data.

### IBC approval

Use of all CRISPR guides and cell lines used were approved by Institutional Biosafety Committee (IBC-University of South Florida: PROTO2022–041) and the guides were used as plasmids instead of viruses. No exogenous viruses were used in this study.

### The Cancer Genome Atlas (TCGA) data and CEBPB^High^ and CEBPB^Low^ grouping

LIHC RNA-seq, and miRNA-seq data were from TCGA (https://gdac.broadinstitute.org/) and an integrated patient data sub-set was used, which is based on the patient IDs of the integrated cluster (iC1+iC2+iC3 = 183 samples). The integrated iCluster dataset was based on the expression of 528 signature genes (200+128+200 genes from iC1, iC2, and iC3 respectively) as described previously^8, 55^. The TCGA IDs of iClusters were generously provided by Dr. Lee, Ju-Seog (UT MD Anderson Cancer Center, Houston, TX, USA), Dr. Ronglai Shen (Memorial Sloan Kettering Cancer Center, New York, NY, USA), Dr. David Wheeler (Baylor College of Medicine, Houston, TX, USA) and Dr. Lewis R. Roberts (Mayo Clinic, Rochester, MN, USA). CEBPB expression was then examined based on iClusters and used to classify patients into CEBPB^High^ and CEBPB^Low^ groups (n=20 each to have low signal-to-noise ratio: Figure S1A).

### Cell line, DNA fingerprinting, plasmids, and stable transfections

The cells were maintained as described previously^8^. Briefly, human Hep3B cells (ATCC # HB-8064) were cultured in MEM containing L-Glutamine and Sodium bi-carbonate (Sigma #M4655), with 10% FBS (Sigma#F0926), vitamins (Gibco Life Technologies #11120052), sodium pyruvate (Gibco Life Technologies #11360070), non-essential amino acids (Gibco Life Technologies #11140050), and penicillin-streptomycin (Gibco Life Technologies #15140122). The cells were subjected to STR fingerprinting as per institutional/lab standards. The cells were then expanded, and frozen. Fresh vials were used after every 6 months or after ~25 passages. The cells in culture were tested for mycoplasma periodically using MycoAlert Kit (Lonza).

The lentiviral constructs were described previously^8^. Briefly, glycerol stocks of mammalian expression vectors such as the control pLenti-GIII-CMV-RFP-2A-Puro (Cat# LV084) and CEBPB pLenti-GIII-CMV-human-CEBPB-RFP-2A-Puro (corresponding to the LAP isoform) (Cat# LV796074) were purchased from Applied Biological Materials Inc., Richmond, BC, Canada. The plasmids were isolated using plasmid MIDIprep kit (Qiagen). The lentiviral expression cassettes were used as plasmids for transfection rather than as viruses or with accompanying plasmids to package viruses, because C19MC is a cluster that responds to viral infections. CRISPR plasmids constructed were described under specific sections below. All plasmids were isolated using Qiagen MIDI prep kit (#12143).

Hep3B cells were stably transfected using control and CEBPB plasmids (not viruses in the case of lentiviral plasmids) and Lipofectamine 2000 (Life Technologies # 11668019) as described previously^8^.

For CRISPR stable cells please see the section below: CRISPR genetic engineering of human cells

### Chemical reagents/inhibitors and usage

A list of chemical agents used in this study is provided in **Table S2**.

### Enzymes, kits, antibodies, transfection reagents and usage

A list of protein and antibody reagents used in this study are provided in the **Table S3**.

### Evaluation of CEBPB-LIP versus CEBPB-LAP expression in CEBPB^High^ and CEBPB^Low^ groups

The TCGA RNA-seq data BAM files were accessed from dbGAP. NIH database of Genotypes and Phenotypes (dbGAP: The results published here are in part based upon data generated by The Cancer Genome Atlas managed by the NCI and NHGRI. Information about TCGA can be found at http://cancergenome.nih.gov). CEBPB^High^ and CEBPB^Low^ files were loaded and visualized in Integrative Genomics Viewer (IGV: BROAD institute, version 2.4.10). The data range was kept constant for all patient sample tracks, but different colors were assigned for CEBPB^High^ versus CEBPB^Low^ tracks. The CEBPB locus covering both LIP and LAP isoforms (chr20:48,806,988–48,809,360, hg19) was focused to show the relative abundance of CEBPB-LIP and LAP isoforms in CEBPB^High^ group compared to CEBPB^Low^ group (Figure S1B). Note, the reads do not fall into the CEBPB-AS1 region (beginning of the focus frame, and hence, the reads are specific to CEBPB-LAP but not to CEBPB-AS1). The image was exported and composited in Adobe Photoshop CS5 (Adobe Systems Inc., San Jose, CA, USA).

### Two-way Gene set Enrichment Analyses (GSEAs) profiling of CEBPB^High^ vs. CEBPB^Low^ patient RNA-seq data and visualization in R

Two-way GSEA was performed as described previously^35^. Briefly, the RNA-seq data of CEBPB^High^ and CEBPB^Low^ groups of TCGA-LIHC were subjected to FPKM adjustment and group averages for all individual genes were calculated. The group averages were subjected to GSEA analyses for C5-Gene ontology complete gene-set collection/module (MsigDB: https://www.gsea-msigdb.org/gsea/msigdb/ version 7.2). The phenotypes analyzed were CEBPB^High^ and CEBPB^Low^ tumors and CEBPB^Low^ and CEBPB^High^ tumors (switching Class-A versus Class-B phenotype and vice versa) and the 7932 normalized enrichment scores (NES) and FDRq values were matched between each GSEA analyses and top contrasting ranks were calculated based on NES. Top-ranked contrasting gene-sets with the lowest FDRq-value in CEBPB^High^ and CEBPB^Low^ groups were categorized and used to generate dot plots in R using NES and corresponding FDRq value. Dot plots (Figures 1A, 3A, 3C, and 5A) were generated in R using ggplot2 package^56^ (R version 4.1.3).

R code

> ggplot(dfName, aes(x = Xgroup, y = YGeneset)) +

geom_point(aes(size = NegFDRq, color = NES)) +

geom_point(shape=0, size=12)+

scale_color_gradientn(colours = c(“black”, “red”, “white”, “navy”, “magenta”),

limits = c(−3.5, 3.5),

breaks = c(−3.00, −2.00, −1.00, 0, 1.00, 2.00, 3.00))+

theme_classic()

# Limits kept constant for all CEBPB^High^ vs. CEBPB^Low^ dot plots

# NegFDRq: The FDRq values converted to negative integers to reflect false discoveries as smaller dots.

### C19MC^High^ vs. C19MC^Low^ Gene set Enrichment Analyses (GSEAs) in 20 different cancer types and visualizations in R

The data of C19MC^High^ and C19MC^Low^ groups of 20 cancer types that had C19MC expression values above or below the cut-off value were previously subjected to one-way GSEA analysis^37^. We selected gene set enrichments from C19MC^High^ vs. C19MC^Low^ dataset that matches/related to the CEBPB^High^ vs. CEBPB^Low^ two-way GSEA top candidate signatures (described above) and generated dot plot (Figure 6A) in R using the ggplot2 package^56^ (R version 4.1.3).

R code

> ggplot(dfName, aes(x = Xgroup, y = YGeneset)) +

geom_point(aes(size = NegFDRq, color = NES)) +

geom_point(shape=0, size=12)+

scale_color_gradientn(colours = c(“black”, “red”, “white”, “navy”, “magenta”),

limits = c(−2.5, 2.5),

breaks = c(−2.00, −1.00, 0, 1.00, 2.00))+

theme_classic()

# NegFDRq: The FDRq values converted to negative integers to reflect false discoveries as smaller dots.

### Copy number analysis of human genome and chromosome-8 in CEBPB^High^ vs. CEBPB^Low^ patients and EnrichR analysis

Human genome-wide and chromosome-8 copy number in CEBPB^High^ vs. CEBPB^Low^ patients were analyzed using TCGA-LIHC copy number variation data^57^ (Figure S1C) within the cBioportal platform with built-in IGV visualization option^58^. The default color code applies to the copy number alterations. A minor increase in CNA was observed in chromosome-8q, and hence chromosome-8 was further focused (Figure S1D) to get the genes that exhibit increased CNA in chromosome-8q using cBioportal and the 44 genes that have significant (p-value <0.01) differential expression between CEBPB^High^ and CEBPB^Low^ patients were fed into EnrichR^59–61^. The results from GO:BP, GO:CC, REACTOME, KEGG, and Bioplanet modules that corroborate with GSEA data were composited in Adobe Photoshop CS5 (Adobe Systems Inc., San Jose, CA, USA) (Figure 1J).

### Shared gene signature analysis: Mitochondria^obesity/neurodegeneration^ (M^on^)-gene-signature

GO:MF Oxidoreductase activity on quinolone as acceptor, GO:BP Respiratory electron transport chain, and GO:BP Oxidative phosphorylation signatures were obtained from MSigDB. All three signatures were fed into GeneVenn (https://www.bioinformatics.org/gvenn/) to get shared genes of these signatures. The results were composited in Adobe Photoshop CS5 (Adobe Systems Inc., San Jose, CA, USA) (Figure 1B). Similarly, Mitochondria^obesity/neurodegeneration^ (M^on^)-gene-signature was identified by feeding KEGG: Parkinson’s disease, KEGG: Alzheimer’s disease, Huntington’s disease, Prion disease, and amyotrophic lateral sclerosis (ALS) with other related medical conditions such as non-alcoholic fatty liver disease, diabetic cardiomyopathy and metabolic signatures such as GO:MF Oxidoreductase activity on quinolone as acceptor, mitochondrial electron transport chain complex-I, and tricarboxylic acid (TCA) signatures from MSigDB into GeneVenn (Figure 3D). These signatures were indeed identified from the EnrichR search of the QRF gene set.

### Genomic distribution of quinolone redox family (QRF) genes (Circos):

The genomic locations of QRF genes were visualized as described previously^35^. Briefly, the complete QRF gene list was processed to obtain the (list obtained from MSigDB: GOMF_OXIDOREDUCTASE_ACTIVITY_ACTING_ON_NAD_P_H_QUINONE_OR_SIMILAR_COMPOUND_AS_ACCEPTOR) coordinates from UCSC Genome Browser (Hg19) to examine the genomic location. The genomic coordinates were plotted as circos plot using Circa software (OMGenomics). A combined g-Banding pattern and centromere marking layer is also included (Figure 1C).

### ChIP-seq data analysis: evaluation of CEBPB binding to the TSS of QRF enriched genes

ChIP-seq data analysis was performed as described previously^8^. Briefly, the CEBPB ChIP-seq data was accessed from the Encyclopedia of DNA Elements (ENCODE)^62^. CEBPB ChIP-seq data sets with or without forskolin induction in HepG2 cells [ENCODE: ENCSR000EEX file: ENCFF000XPP was examined for CEBPB binding at transcription start site (TSS) regions of all core enriched quinolone redox family (QRF) genes (2kb up and downstream, hg19) and visualized using Integrative Genomics Viewer (IGV: BROAD institute, version 2.4.10). The data range was kept constant for all core enriched QRF genes. CEBPB binding was represented as heatmaps to examine the binding proximity to the TSS. The genes organized in the negative strand of the genome were flipped direction to represent 5’−3’ representation through TSS in the heatmap composite which was done using Adobe Photoshop CS5 (Adobe Systems Inc., San Jose, CA, USA) (Figure 1D).

### Genetic reagents (plasmid DNAs) and stable cell lines generated.

A list of plasmids generated and or used in this study are provided in the **Table S4**. Usage of viruses was avoided throughout the study because part of the objective is to study the function of anti-viral response cluster C19MC in the absence of exogenous viruses.

### ZNF331-C19MC fusion CRISPR guide RNA cloning and Sanger sequencing.

C19MC often gets translocated to the loci of active genes or to replace CpG86 by chromosomal fusions to regulate its own expression in various pediatric tumors such as undifferentiated embryonal sarcoma of the liver (UESL)^52^ and embryonal tumors with multilayered rosettes (ETMR)^51^. However, it has not been experimentally tested *in vitro* yet. To enhance C19MC expression by a similar mechanism, we chose to engineer the Hep3B cell genome (Chromosome-19) by CRISPR cuts and fusion of C19MC to the 3’-terminal of 3’-UTR of ZNF331 gene, which is the immediate upstream gene with transcription activity (DPRX gene is silent in this cells). We designed CRISPR guide-3, which can cut at the 3’-terminal of 3’-UTR of ZNF331 gene beyond all essential regulatory sequences, and CRISPR guide-4 which can cut just before C19MC but retaining the CEBPB binding site with the C19MC. The CRISPR-guide-3 and 4 were cloned as described previously^37, 63^. Briefly, the plasmid pSpCas9(BB)-2A-Puro (PX459) V2.0 was digested with Bbs-I to release the non-targeting part of the sgRNA, resulting linear vector was gel purified using GFX columns, quantified, and ligated with custom synthesized and annealed oligo duplexes of guide-3 or guide-4 with suggested overhangs using Rapid ligation kit, for overnight incubation at 4° C. The ligated mixtures were transformed into DH5α competent cells with recovery SOC-medium and plated as per standard procedures. Individual colonies were screened by colony PCRs with parallel inoculation for glycerol stock preparation in Ampicillin-LB-broth as described previously^37^. Positive colonies were subjected to plasmid-miniprep and Sanger sequencing (Genewiz/Azenta, USA) using individual “Colony PCR” primers as described previously^37^. Plasmids were confirmed for incorporation of oligonucleotide inserts and then subjected to plasmid MIDI-prep and quantified plasmid DNA before transfections.

### CRISPR genetic engineering in human cells

Step-1: Deleting 85kb and fusing ZNF331 with C19MC:

Hep3B cells were transfected with pSpCas9(BB)-2A-Puro (PX459) V2.0 (has inbuilt SpCas9) or co-transfected with CRISPR-guides 3 and 4 constructs [have inbuilt SpCas9 plus guides targeting the 3’UTR end of ZNF331 (guide 3, Hg19: chr19:54083216, after CEBPB binding site) and just before C19MC and CEBPB binding sites (guide 4, Hg19: chr19:54168944)] using Lipofectamine-2000 as per manufacturer’s protocol with minor modifications (Figure 6B). Transfected cells were stably selected using a gradual increase in puromycin usage from 0.5 mg/ml to 4 mg/ml medium. Upon reaching confluency in transfected cells, the cells were trypsinized and plated for genomic DNA isolation and freezing. Genomic DNA was isolated from SpCas9 control (Nontargeting-Guide-sgRNA pooled stable cells), and ZNF331-C19MC fusion (Guides-3 and 4 co-transfected stable pooled cells) and quantified using Nanodrop. 150 ng of genomic DNAs were subjected to CRISPR screening PCR using “d2DPRX” primers and the amplicon in fusion cells with size ~420 bp (Figure 6B) were confirmed to have undergone deletion of 85-kb region and ZNF331-C19MC fusion of flanking ends, as the primers bind outside the PAM sites.

Step-2: Single cell cloning and Sanger sequencing of CRISPR-engineered regions:

Hep3B-SpCas9, and Hep3B-ZNF331-C19MC fusion cells were plated at 96 well plates in limiting dilution fashion to get single cells per well. Multiple single-cell clones were tested for SpCas9 for control cells and for ZNF331-C19MC fusion in guides-3 & 4 transfected cells. For SpCas9 cells, the cells positive for SpCas9 RNA were chosen and frozen. For fusion, LDC24 single cell colony that had ZNF331-C19MC fusion as well as DPRX gene were chosen. Sanger sequencing was done as described previously. Briefly, 1M betaine in GFX purified PCR bands using d2DPRX individual primers were sent for sequencing to Azenta. Sanger sequencing of these cells revealed that, this single-cell clone has multiple copies of chromosome-19 with both wild-type, ZNF331-C19MC fusion, and another fusion with a single nucleotide shift as ploidy (Figure 6B). We chose this single-cell clone because this means no part of the genome or gene is lost in these cells.

### RT-PCR, genomic DNA-PCRs, bacterial colony PCRs and primers:

A list of PCR primers used in this study and their reaction conditions are provided in the **Table S5**. All PCR reactions were performed with an initial denaturation of 95°C for 3 minutes; cycling conditions of 95°C for 1 minute, 60°C for 30 seconds, 72°C for 1 minute for 34 cycles; and a final extension time of 72°C for 5 minutes. All reactions had a final concentration of 1M betaine.

Reverse transcriptase PCRs

RT-PCRs were performed as described previously^37^. Briefly, total RNA was isolated using TRIZOL reagent (ThermoFisher Scientific #15596026, Waltham, MA, USA) as per manufacturer’s instructions. 20 ml complementary DNA synthesis reactions were done using 1000 ng RNA and High-Capacity cDNA Reverse Transcription Kit (ABI # 4368814, Foster City, CA, USA) with 1.5M final concentration of betaine (from 5M stock: Sigma # B0300–1VL, St. Louis, MO, USA). The temperature conditions were, 25°C for 10m, 37°C for 120m, and 85°C for 5m. The cDNAs were then diluted with 30 ml of nuclease-free water and then 2.5 ml was used per PCR reaction. For PCR reactions 1M betaine (final conc.) was used along with regular PCR reaction components. The primer sequences and obtained product sizes are included in **Table S5**. All PCR reactions were performed with an initial denaturation (95°C) time of 5-minutes, and standardized cycles with a denaturing (95°C) time of 1-minute, annealing temperature of 60°C (30 seconds), and 1 minute of extension time (72°C), with 34 cycles, and a final extension (72°C) time of 5-minutes. The PCR reactions were run on 2% agarose gels with GeneRuler 100 bp DNA Ladder (ThermoFisher Scientific #SM0243). The gels were imaged using LI-COR Odyssey Fc imager (Lincoln, NE, USA).

### Immunofluorescence and quantifications

NDUFA3:

Immunofluorescence was performed as described previously^26, 37, 64^. Briefly, stable Hep3B pGIII-control cells (RFP expressing), Hep3B pGIII-CEBPB-LAP cells (RFP expressing), or CRISPR-engineered Hep3B-SpCas9-control cells and Hep3B-ZNF331-C19MC-fusion cells were plated in 24 well plates at 50,000 cells/ml and cultured for 48hrs with a media change at 24hrs. For some experiments, the cells were treated with 20 mM chloroquine (CQ) for 24 hours. The medium was removed, and the cells were fixed using ice-cold methanol at −20°C for a period of 24–48hrs. Then, the cells were washed with PBS thrice and blocked using 300 ml blocking buffer/well (1% BSA and 0.3% Triton-X100 in PBS) for 30 minutes at room temperature. Then, the cells were incubated with NDUFA3 (B-12) mouse monoclonal antibody (Santacruz Biotechnology, Sc-365351: Dilution 1:200) in blocking buffer for overnight at 4°C. The cells were washed with PBS and incubated with Goat anti-Mouse IgG (H+L) Cross-Adsorbed Secondary Antibody, Cy5 labeled, 1:1000 dilution in blocking buffer (A10524: Life Technologies) for 1 hour at room temperature with light-protected mode from this step onwards. The cells were washed with PBS thrice and incubated with 10 nM Hoechst-33342 in PBS (Sigma) for 20 minutes, and imaged. Exposure times for each channel were kept constant between conditions.

The fluorescence signal per cell was quantified using ImageJ to calculate the average NDUFA3 expression/cell. 200 cells from replicates were measured per condition and the data were plotted as an aligned dot plot with standard error of the mean in GraphPad Prism software. The plots were composited in Adobe Photoshop CS5 (Adobe Systems Inc., San Jose, CA, USA) (Figures 1E, 1H, and 6G). The mean values were indicated in the plots.

Nuclear Notch1 intracellular domain (nNICD):

Immunofluorescence for Notch1 was performed as described above for NDUFA3 in stable Hep3B pGIII-control cells (RFP expressing), Hep3B pGIII-CEBPB-LAP cells (RFP expressing), and CRISPR-engineered Hep3B-SpCas9-control cells, and Hep3B-ZNF331-C19MC-fusion cells, but with primary Notch1 (A-8) mouse monoclonal antibody (Santacruz Biotechnology, Sc-376403: Dilution 1:200).

The nuclei and Notch1 fluorescence were imaged as individual channels, and the nuclear boundaries were determined using magic wand selection tool in Adobe Photoshop CS5 (Adobe Systems Inc., San Jose, CA, USA), and the selection was inverted. The inverted selection was transferred in place to the Notch1 channel image, and deleted the cytoplasmic Notch1 and background fluorescence signals (as the selection is inverted nuclear selection), leaving only nuclear notch1 (nNICD) fluorescence signal. The nuclear NICD (nNICD) fluorescence signals per individual nuclei were then quantified using ImageJ free-hand selection tool to further calculate average nuclear NICD localization/cell. 200 cells from replicates were measured per condition, and the data were plotted as aligned dot plot with standard error of the mean in GraphPad Prism software or visualized using Anaconda/python/Jupyter environment (Code below). The plots were composited in Adobe Photoshop CS5 (Adobe Systems Inc., San Jose, CA, USA) (Figures 4A, and 7A). The mean values were indicated in the plots.

Anaconda/Jupyter/Python code

[1]: import seaborn as sns

[2]: sns.__version__

‘0.12.2’

[3]: import pandas as pd

[4]: df=pd.read_csv(‘dfName.csv’)

[5]: df

[6]: sns.set_style(“whitegrid”)

g=sns.jointplot(kind=‘scatter’, data=df, x=‘NICDintensityDiv100’, y=‘NucAreaDiv100’,

hue=‘CellTreatmentGroup’,

palette = [“#0cdd0c”, “#030000”],

legend=False)

# df: data frame; dfName: data frame name; Legend=False was used to omit legends after identifying the group colors.

### Reactive oxygen species (ROS) imaging, quantification and visualization

Baseline ROS imaging was performed as described previously^20^. Briefly, Hep3B-pGIII, Hep3B-CEBPB-LAP overexpressed cells or Hep3B-SpCas9 control, Hep3B-ZNF331-C19MC fusion CRISPR-engineered cells were plated at a density of 50,000 cells/ml with 4 ml of cells/well onto six-well plates. 24 hours after plating, the cells were given a complete medium change and further incubated for 24 hours. 30 minutes before ending incubation, 10 μM DCF-DA was added to all wells, and cells were incubated for a further 30 minutes and subjected to imaging ROSactivated DCF-DA by fluorescence microscopy with constant exposure settings between conditions.

DCF-DA fluorescence per cells were quantified using ImageJ referencing bright field images to justify cell boundaries including podosomal belts. Two hundred quantifications were performed per condition from replicates, and the data were plotted as aligned dot plot with standard error of the mean in GraphPad Prism software, or visualized in Anaconda/Python/Jupyter environment (code below). The plots were composited in Adobe Photoshop CS5 (Adobe Systems Inc., San Jose, CA, USA) (Figures 1F, and 6H). The mean values were indicated in the plots. Tests of significance were done using Microsoft Excel 2021.

Anaconda/Jupyter/Python code

[1]: import seaborn as sns

[2]: sns.__version__

‘0.12.2’

[3]: import pandas as pd

[4]: df=pd.read_csv(‘dfName.csv’)

[5]: df

[6]: sns.set_style(“whitegrid”)

g=sns.jointplot(kind=‘scatter’, data=df, x=‘ROSDiv1000’, y=‘CellSizeDiv100’,

hue=‘Group’,

palette = [“#030000”,”#07b7e8”,],

legend=False)

# df: data frame; dfName: data frame name; Legend=False was used to omit legends after identifying the group colors.

### Podosomal belt quantifications

Hep3B-pGIII, Hep3B-CEBPB-LAP overexpressed cells were plated at a density of 50,000 cells/ml with 4 ml of cells/well onto six-well plates. 24 hours after plating, the cells were given a complete medium change and further incubated for 24 hours or treated as indicated in figures for 24 hours. Random images were taken from replicates using bright field settings and the number of cells with podosomal belts. The percentage of podosomal belt positive cells at baseline was then calculated based on the total number of cells per field, and the SEM values were plotted in GraphPad Prism software. The plots were composited in Adobe Photoshop CS5 (Adobe Systems Inc., San Jose, CA, USA) (Figures 1G and 2F). The mean values were indicated in the plots. Tests of significance were done using Microsoft Excel 2021. Alternatively, the control values were considered as 1, and the relative fold change was calculated and represented to show the increase or decrease in podosomal belt formation in response to treatments (Figure 4C).

### Notch-1, mTOR, Src, A-to-I RNA-editing, autophagy, NMD inhibitor experiments

Hep3B-pGIII, Hep3B-CEBPB-LAP overexpressed cells or Hep3B-SpCas9 control, Hep3B-ZNF331-C19MC fusion CRISPR-engineered cells were plated at a density of 50,000 cells/ml with 4 ml of cells/well onto six-well plates. 24 hours after plating, the cells were treated with chloroquine (CQ), hemin, MK0752, dasatinib, Torin1, 8-AzaAdenosine, or caffeine (doses were indicated in **Table S2**) in complete medium and further incubated for 24 hours at 37° with 5% CO_2_. The cells were then further processed as per specific methods described in this study including immunofluorescence, podosomal belt quantification, vacuole quantification, lipid droplet quantification, RT-PCRs, nNICD quantification, time-lapse microscopy, ROS quantification, RNA-editing and so on.

### Baseline mitochondrial load quantification

The baseline mitochondrial load per frame was quantified as described previously instead of mitochondrial load per cell^37^. Briefly, Hep3B-SpCas9 control, and CRISPR-engineered Hep3B-ZNF331-C19MC fusion cells were plated at a density of 50,000 cells/ml with 4 ml of cells/well onto six-well plates. 24 hours after plating, the cells were treated with 30 nM of Cy5-CSi000A and incubated for 24 hours at 37° with 5% CO_2_ with light protection. The cells were then imaged using constant exposure settings to image Cy5-CSi000A stained mitochondria. The total fluorescence per frame was divided by the total number of cells per frame. The control values were considered as 1, and the relative change in response to CRISPR human genetic engineering enabled C19MC miRNA expression (ZNF331-C19MC fusion) was represented as fold change in aligned dot plot with SEM in GraphPad Prism software. The plots were composited in Adobe Photoshop CS5 (Adobe Systems Inc., San Jose, CA, USA) (Figure 6H). The mean values were indicated in the plots. Tests of significance were done using Microsoft Excel 2021.

### Lipid droplet imaging, quantification, and visualizations

Hep3B-pGIII, Hep3B-CEBPB-LAP overexpressed cells or Hep3B-SpCas9 control, Hep3B-ZNF331-C19MC fusion CRISPR-engineered cells were plated at a density of 50,000 cells/ml (4 ml of cells/well) onto six-well plates. 24 hours after plating, the cells were treated with chloroquine (CQ), with or without hemin for 24 hours. Nile red was added to all cells at a dose of 1mg/ml during the treatment stage and protected from light while on incubation at 37° with 5% CO_2_. The cells were then imaged at constant exposure settings using Zeiss Observer.Z1 microscope equipped with Axiocam 503 mono (Zeiss) camera. Individual channel images were taken, and the lipid droplet (LD) fluorescence was pseudo-colored to red, merged, and exported to jpeg using ZEN 2.3 Pro software (Carl Zeiss Microscopy, GmbH, 2011, Blue edition). The final composite was created using Adobe Photoshop CS5 (Adobe Systems Inc., San Jose, CA, USA).

Using ImageJ, individual cell lipid droplet fluorescence was quantified in addition to cell area from replicates, and the data were plotted as an aligned dot plot with SEM of the lipid droplet fluorescence in GraphPad Prism software. The plots were composited in Adobe Photoshop CS5 (Adobe Systems Inc., San Jose, CA, USA) (Figure 7B). The mean values were indicated in the plots. Tests of significance were done using Microsoft Excel 2021. Alternatively, the lipid droplet fluorescence, and cell area data were subjected to transformation by dividing the arbitrary ImageJ units (AU) with 1000 or 100 respectively and subjected to XY scatter plotting in jointplot format (Figure 3B) using the python code below.

Anaconda/Jupyter/Python code

[1]: import seaborn as sns

[2]: sns.__version__

‘0.12.2’

[3]: import pandas as pd

[4]: df=pd.read_csv(‘dfName.csv’)

[5]: df

[6]: sns.set_style(“whitegrid”)

g=sns.jointplot(kind=‘scatter’, data=df, x=‘LipidDropletIntensityDiv1000’, y=‘CellAreaDiv100’,

hue=‘CellTreatmentGroup’,

palette=‘rocket’,

legend=False)

# df: data frame; dfName: data frame name; Legend=False was used to omit legends after identifying the group colors.

### Quantitative microscopy, time-lapse microscopy, and data visualization in R and Python

Microscopy was performed as described previously^8, 36^. Cells were imaged using Zeiss Observer.Z1 microscope equipped with Axiocam 503 mono (Zeiss) camera. For fluorescence microscopy the individual channel images were pseudo-colored if indicated in figures/legends, merged, and exported to jpeg using ZEN 2.3 Pro software (Carl Zeiss Microscopy, GmbH, 2011, Blue edition). The final composite was created using Adobe Photoshop CS5 (Adobe Systems Inc., San Jose, CA, USA).

For immunofluorescence or lipid droplet quantifications, either individual cells or nuclei were marked using ImageJ, and the intensities were quantified. For vacuole area and translucency quantifications, the bright field images were threshold adjusted so as to mark the translucent areas into dark areas before quantifying it using particle measurement tool. A gate of <100 AU was used to exclude artifacts, and the large translucent vacuoles of >100 AU size were taken into account for measurements.

Podosomal belts with or without membrane ruffles were focused, and then the time-lapse microscopy was performed using the constant exposure settings at a frame rate of 30 seconds or, 5 or 6 minutes per frame. All time-lapse microscopies were done under 30 minutes overall duration, as the podosomal belt collapse does not exceed 30 minutes. Multiple time-lapse imaging experiments were done to differentiate vacuole forming podosomal belt collapse (CQ treated conditions) versus non-vacuole forming podosomal belt retraction (untreated conditions).

The individual cell measurement data were either visualized using R, or Python/Anaconda/Jupyter environment. The codes are given under appropriate sections.

### Quantitative real-time PCRs

RNAs were isolated from Hep3B parental cells, Hep3B-SpCas9, and Hep3B-ZNF331-C19MC fusion cells using miRNeasy Mini Kit (Qiagen #217004, Germantown, MD, USA). RNAs were quantified using Nanodrop, and 250 ng RNAs were subjected to cDNA synthesis using Multiscribe reverse transcriptase with RNAse inhibitor, 10X buffer, dNTPs, (ABI, Cat # 4366596) and RT TaqMan^™^ Primers (RNU6B Control Assay: Assay ID: 001093 (Cat # 4427975), hsa-miR-520g-3p: Assay ID: 001121 (Cat # 4427975). The cDNAs were then subjected to real-time PCR reactions in triplicates using respective primers with probes and TaqMan master mix. The data were normalized using RNU6B, and the comparative Ct (ΔΔCt) method was used to compute the relative expression of miRNAs after normalizing with RNU6B values. Hep3B-SpCas9 cells were omitted from the analysis as the C19MC expression was suppressed enough to give undetermined values. Therefore, a comparison of Hep3B-ZNF331-C19MC fusion cells was made with the parental cells. Statistical significance was calculated in Microsoft Excel (2021) using t-test, two-tailed distribution, and two-sample unequal variation option. The results and standard error of the mean (SEM) were then plotted using GraphPad Prism software (v7.04; La Jolla, CA, USA).

### RNA-seq, EnrichR, and Network analysis

RNAs from stable Hep3B-SpCas9 and Hep3B-ZNF331-C19MC fusion cells were isolated using miRNeasy Mini Kit (Qiagen #217004, Germantown, MD, USA), with an on-column RNAse-free DNAse (Qiagen # 79254) digestion as per manufacturer’s protocol. RNA-seq was then performed in quality control tested RNAs using the NuGen Ovation RNA-seq FFPE System (PN 7150–08) to prepare the libraries and were run on the Illumina NextSeq 500 with a 76-base paired-end read. The adapter reads were trimmed using Cutadapt (v1.8.1), and raw reads were then aligned to the human genome (build: hg19) using STAR (v2.5.3a)^65^. Gene expression was evaluated as read count at the gene level with HTSeq (v0.6.1)^66^ and Gencode gene model v28. Gene expression data were then normalized using DEseq2^67^. The data was composited in Adobe Photoshop CS5 (Adobe Systems Inc., San Jose, CA, USA).

### 3D graphics

3D chromosomes, 3D DNA images were generated using Lightwave Modeler v11.6.3 and rendered using Lightwave Layout v11.6.3 (NewTek Lightwave San Antonio, TX, USA) and composited using Adobe Photoshop CS5. Other graphic images were created using Adobe Photoshop CS5 (Adobe Systems Inc., San Jose, CA, USA).

### RNA secondary structure prediction, cDNA-preparation, A-to-I RNA-editing, and Sanger sequencing

While the DEPTOR^46^ and HBV RNAs^45^ are known for RNA-editing, we examined novel sites within these mRNAs that comply with sequence requirements and predicted secondary structure for efficient editing. We chose HBV-polymerase RNA as it has a pivotal and potential role in transcribing HBV genes. We fed the selected exon-4 (Transcript variant-1, GenBank: NM_022783.4) (in the case of DEPTOR) and multi-strain (15 strains) conserved (in the case of HBV-polymerase) sequences into trRosettaRNA web-server^68^. Secondary structure prediction was considered even if it is low confidence as the A-to-I RNA-editing sequence-based nucleotide combinations were manually examined for additional confirmation before experiments.

Total RNAs were isolated using Trizol reagent from Hep3B-pGIII, Hep3B-CEBPB-LAP overexpressed cells or Hep3B-SpCas9 control, Hep3B-ZNF331-C19MC fusion CRISPR-engineered cells treated with or without reagents indicated in the figures. The RNAs were converted to cDNAs, and PCRs were performed as described in the RT-PCR section. The bands were gel purified using GFX columns and 5–10ng of purified DNAs were subjected to Sanger sequencing (Azenta) with 1M betaine, single forward or reverse primer included as final concentration in the pre-sequencing mix. For each experimental batch, the template and primer concentrations were kept constant.

The sequencing results were processed using SnapGene viewer with constant X and Y magnification settings to identify the level of RNA-editings which replace adenosine with thymidine compared to the wild type sequence. The chromatograms were screenshot snippet copied in Windows 10 and composited in Adobe Photoshop CS5 (Adobe Systems Inc., San Jose, CA, USA).

### Softwares, packages, web-servers used and figure Compositing

A list of softwares used in this study is listed in **Table S6**. All graphs, plots, and images exported from respective softwares were finally composited in Adobe Photoshop CS5 while keeping the main plot area original but replacing axis fonts to have uniform font size. Labeling, and mean (dotted) lines were introduced in photoshop (Figure 2A, 2G), or in GraphPad Prism software. Certain elements were added to the microscope image to mark the boundary of the nucleus, which was complemented with unmarked panels (Figure 4A nuclear boundary white line, Figure 8 annotated panels). Arrows, arrowheads, and scale bar lines of magnification of microscope images were also added using Photoshop in the composite, where the scale bars are based on the original microscope image scale bars.

### Statistical analyses

Frequency distribution aligned dot-plots and statistical analyses were done using GraphPad Prism software (v7.04; La Jolla, CA, USA). The data points are shaded to 50% transparency to reveal the density. For GSEA, NES and FDRq values less than 0.1 were considered significant. Throughout the study, the p-value of 0.05 was considered significant, and p-values <0.001 were considered as robust significance. The ‘n’ for individual cell quantification experiments were kept constant, which were 200 observations from three replicates to show the overall distribution of the data range. All statistical data were represented as standard error of the mean (S.E.M.), and all p-values were directly indicated within each figure panels. For all plots, the t-test p-values were calculated in Microsoft Excel 2021.

## Supplementary Files

This is a list of supplementary files associated with this preprint. Click to download.

- 000SupplementaryinformationSubmission.docx
- 0SuppFigure1.jpg
- Supp.docx

## Data Availability

This paper does not report original codes but uses modified codes, which are given under the appropriate methods sections below. This paper uses public TCGA data sets, and details are given below under the appropriate methods sections.
